# MRI-based radiomics models for the early prediction of radiation-induced temporal lobe injury in nasopharyngeal carcinoma

**DOI:** 10.3389/fneur.2023.1135978

**Published:** 2023-03-16

**Authors:** Lixuan Huang, Zongxiang Yang, Zisan Zeng, Hao Ren, Muliang Jiang, Yao Hu, Yifan Xu, Huiting Zhang, Kun Ma, Liling Long

**Affiliations:** ^1^Department of Radiology, The First Affiliated Hospital of Guangxi Medical University, Nanning, Guangxi, China; ^2^Department of Radiology, The Second Affiliated Hospital of Guangxi Medical University, Nanning, Guangxi, China; ^3^MR Scientific Marketing, Siemens Healthineers Ltd., Wuhan, China; ^4^CT Imaging Research Center, GE Healthcare China, Guangzhou, China; ^5^Key Laboratory of Early Prevention and Treatment for Regional High Frequency Tumor, Gaungxi Medical University, Ministry of Education, Nanning, Guangxi, China; ^6^Guangxi Key Laboratory of Immunology and Metabolism for Liver Diseases, Nanning, Guangxi, China

**Keywords:** radiation-induced temporal lobe injury, nasopharyngeal carcinoma, radiomics, magnetic resonance imaging, prediction

## Abstract

**Objective:**

This study was conducted to develop and validate a radiomics-clinics combined model-based magnetic resonance imaging (MRI) radiomics and clinical features for the early prediction of radiation-induced temporal lobe injury (RTLI) in patients with nasopharyngeal carcinoma (NPC).

**Methods:**

This retrospective study was conducted using data from 130 patients with NPC (80 patients with and 50 patients without RTLI) who received radiotherapy. Cases were assigned randomly to training (*n* = 91) and testing (*n* = 39) datasets. Data on 168 medial temporal lobe texture features were extracted from T1WI, T2WI, and T1WI-CE MRI sequences obtained at the end of radiotherapy courses. Clinics, radiomics, and radiomics–clinics combined models (based on selected radiomics signatures and clinical factors) were constructed using machine learning software. Univariate logistic regression analysis was performed to identify independent clinical factors. The area under the ROC curve (AUC) was performed to evaluate the performance of three models. A nomogram, decision curves, and calibration curves were used to assess the performance of the combined model.

**Results:**

Six texture features and three independent clinical factors associated significantly with RTLI were used to build the combined model. The AUCs for the combined and radiomics models were 0.962 [95% confidence interval (CI), 0.9306–0.9939] and 0.904 (95% CI, 0.8431–0.9651), respectively, for the training cohort and 0.947 (95% CI, 0.8841–1.0000) and 0.891 (95% CI, 0.7903–0.9930), respectively, for the testing cohort. All of these values exceeded those for the clinics model (AUC = 0.809 and 0.713 for the training and testing cohorts, respectively). Decision curve analysis showed that the combined model had a good corrective effect.

**Conclusion:**

The radiomics–clinics combined model developed in this study showed good performance for predicting RTLI in patients with NPC.

## Introduction

Most NPCs are highly sensitive to radiotherapy, and radiotherapy has become the first choice for the treatment of nasopharyngeal carcinoma (NPC) (1). Although radiotherapy technology for patients with NPC has been greatly improved in recent years, associated complications after the treatment are still common in clinical practice, such as radiation-induced temporal lobe injury (RTLI) (2, 3). Due to the anatomical location of NPC, the target radiotherapy volume inevitably involves the medial temporal lobe. RTLI is the most likely neurological complication following radiotherapy for NPC (4), and its influence on patients' quality of life far exceeds that of the tumor itself. The focus of tumor treatment has expanded from the improvement of the survival rate to the prevention and treatment of tumor-related complications. The American Cancer Society Center has emphasized that the prevention and treatment of complications following comprehensive cancer treatment should be a top research priority to maximize patients' quality of life (5). The detection of RTLI in the early stage enables its effective treatment. Therefore, RTLI has received increasing attention, and the prediction of RTLI early in the incubation period is of decisive significance in the final prognosis.

According to the timing of clinical symptom onset after radiotherapy, radiation brain injury is classified as an acute reaction period, early-delayed period, and late-delayed period (6). In the acute reaction period (a few days to a few weeks after radiotherapy), patients may have no symptoms or only elevated intracranial pressure. Pathological changes mainly include increased vascular permeability, inflammatory cell infiltration, brain tissue congestion, and edema. The early-delayed period (1–6 months after radiotherapy) is characterized by the demyelination of glial cells and axonal edema. Complete recovery can be achieved in these first two stages with active clinical treatment. In the late-delayed period (6 months to several years after radiotherapy), the pathological manifestation is radiation necrosis, namely, radiation encephalopathy (REP). Brain tissue damage has been progressive and is irreversible at this stage (7).

Currently, RTLI is mainly diagnosed by imaging examination, with auxiliary cognitive function assessment (8, 9). Computed tomography (CT) and conventional MRI are the main modalities used, but their diagnostic ability is not ideal (10). They enable the diagnosis and staging of brain injury in the late reaction stage, when it is often severe and cannot be treated effectively, which greatly affects patients' survival and quality of life. The study of the pre-symptomatic phase is clinically more important than the study of the symptomatic or clinical phase. More-sensitive imaging and analytical techniques enable the detection of early tissue changes and the prediction of outcomes, enabling the implementation of adequate neuroprotective or other preventive treatments (11). In recent years, functional imaging techniques such as magnetic resonance spectroscopy (MRS), diffusion-weighted imaging (DWI), diffusion tensor imaging (DTI), and dynamic contrast enhancement (DCE) have been used to complement routine MRI and provide functional and metabolic information (12–15). However, the inclusion of functional MRI in routine scanning protocols is often not possible due to the high equipment requirement. In addition, the selection of the same voxel sites for analysis in the same patient on repeat examination cannot be guaranteed with the use of these modalities. Given spatial resolution limitations, the detection of subtle changes in white matter structure *via* the application of regional spatial statistics is difficult (16). Thus, an effective method for the early prediction of RTLI development is needed.

Radiomics is the process by which medical images are transformed into high-dimensional measurable data through the high-throughput extraction of quantitative features, which are then analyzed to support decision-making (17). The analysis of these features is of great value for the classification, efficacious evaluation, and prediction of the prognosis of many diseases, including RTLI. Radiomics can provide information about microstructural changes in the temporal lobe that are invisible to the naked eye, which may serve as biomarkers for the early prediction of RTLI. Zhang et al. (16) developed three radiomics models to predict RTLI based on follow-up MRI scan [T1-weighted imaging (T1WI-CE) and T2-weighted imaging (T2WI) sequences] in the last 3 years before RTLI confirmation based on MRI scan. They found that these three radiomics models enable the early dynamic prediction of RTLI.

Due to the regional characteristics of NPC, few large-scale comprehensive studies of early brain changes and RTLI prediction in patients with the disease have been performed. The purpose of this study was to investigate the role of the radiomics–clinics combined model-based magnetic resonance imaging (MRI) radiomics and clinical features for the early prediction of RTLI in patients with NPC and to provide a basis for clinician decision-making to prevent or slow the deterioration of RTLI.

## Materials and methods

### Patients

The Ethics Committee of the First Affiliated Hospital of Guanxi Medical University, China, approved this retrospective study (no. 2022-E416-01) and waived the requirement for informed consent. Data from 130 patients with NPC (80 patients with and 50 patients without RTLI) who received radiotherapy between January 2010 and July 2022 were included. The inclusion criteria were as follows: (1) pathological confirmation of NPC; (2) receipt of intensity-modulated radiation therapy (IMRT); (3) MRI examination before treatment and within 1 week after the end of radiotherapy course; (4) patients with RTLI, confirmation of RTLI based on MRI; and 5) patients without RTLI, with follow-up time >108 months and confirmation based on MRI. The exclusion criteria of our study were as follows: (1) with primary temporal lobe invasion, other intracranial tumors, or cerebrovascular disease; (2) without regular follow-up; (3) incomplete MRI and clinical data; (4) poor MRI image quality.

Intensity-modulated radiation therapy (IMRT) was planned and implemented using the Eclipse system (Varian Medical Systems, Palo Alto, CA, USA). The target area and dose design of IMRT for NPC were based on the expert consensus of the Radiation Treatment Oncology Organization (RTOG). The radiation doses of Nasopharynx gross tumor volume (GTVnx), lymph node gross tumor volume (GTVnd), clinical target volume-1 (CTV-1), and clinical target volume-2 (CTV-2) were 70–72, 64–72, 62–64, and 54–56 Gy, respectively, 30–33 f, five times a week for 6–7 weeks. Clinical data collected were the patient's age and gender, WHO pathological type, clinical stage, TN stage, radiation dose, and chemotherapy receipt.

### Diagnostic criteria and follow-up of RTLI

Radiation-induced temporal lobe injury (RTLI) was diagnosed when at least one of the following three manifestations was detected on MRI: (1) white matter lesions [(WMLs) homogeneously high signal intensity on T2WI and low signal intensity on T1-weighted imaging (T1WI)], (2) contrast-enhanced lesions (high signal intensity on T2WI and postcontrast enhancement on T1WI), and (3) cysts (round lesions with thin or imperceptible walls and very high signal intensity on T2WI) (18). Differential diagnosis was performed to ensure that these features were not caused by other factors, such as tumor metastasis.

According to the guidelines of the National Comprehensive Cancer Network, patients underwent follow-up assessment (physical and MRI examination) every 3 months for the first year after the completion of radiotherapy, every 6 months for the second year, and annually thereafter until RTLI was detected (19). Two radiologists independently examined the patients' clinical records and MR images, with any disagreement on the data recorded resolved through discussion.

### MRI acquisition

All patients underwent an MRI examination with a 1.5 Tesla scanner (GE Signa Echospeed; GE Medical Systems, Milwaukee, WI, USA). T1WI, T2WI, and T1WI-CE were performed with the same geometric parameters. The main parameters included: field of view = 240 mm × 230 mm; spacing between slices = 1 mm; slice thickness = 5 mm; matrix = 232 × 219; spatial resolution = 0.25 mm × 0.25 mm × 5.0 mm; and repetition time (TR)/echo time (TE) = 660/14 ms for T1WI, 6000/70 ms for T2WI, and 550/12 ms for T1WI-CE. Gadolinium-diethylenetriamine penta-acetic acid (0.1 mmol/kg body weight, Magnevist; Schering Diagnostics AG, Berlin, Germany) was used as the contrast agent and injected at a rate of 2 mL/s.

### MR image segmentation and radiomics feature extraction

We exported the MR images from the PACS system to the ITK-SNAP software (version 3.6.0; www.itksnap.org) for image segmentation. These were key points for image segmentation: regions of interest (ROIs) were drawn transversely along the borders of each layer in the mid-inferior level of the temporal lobe (extending from the level of the cerebral peduncles to the level at which the temporal lobe disappeared, including white and gray matter) while avoiding sulci and fissures. Cases of RTLI that first occurred unilaterally were outlined on the affected side and those developed bilaterally were outlined on the left side.

A radiologist with 10 years of experience in the diagnosis of head and neck disease delineated three-dimensional volume of interest (VOI) in the mid-inferior temporal lobe layer by layer on T2W images; a senior physician with more than 20 years of experience confirmed the VOIs. Both professionals were blinded to the patient's clinical data. The texture features were extracted from the sequences of T1WI, T2WI, and T1WI-CE using FeAture Explorer Pro (FAE, version 0.5.1) in Python (version 3.7.6) (20). These features included the original shape, first order, and gray-level co-occurrence matrix (GLCM).

### Feature selection and radiomics signature building

In total, 168 radiomics features were extracted for each patient. Clinical features included the age and gender of patients, WHO pathological type, clinical stage, TN stage, radiation dose, and chemotherapy receipt. We developed clinics, radiomics, and radiomics–clinics combined models for RTLI prediction in the training and testing cohort, including all clinical factors, 168 radiomics features, and selected radiomics signatures and clinical factors, respectively. We labeled the RTLI-negative group as 0 and the RTLI-positive group as 1. Computer-generated random numbers were used to assign 70% of the cases (*n* = 91; 56/35 = positive/negative) to the training dataset and the remaining cases (*n* = 39, 24/15 = positive/negative) to the testing dataset.

To remove the unbalance of the training dataset, we up-samples by repeating random cases to make positive/negative samples balance. To build the scout model, we applied normalization to the feature matrix. The Eigenvector was subtracted from the mean value of the vector and divided by its length. Due to the high dimensionality of the feature spaces and the need to obtain independent features, Pearson correlation coefficient (PCC) values were calculated between all feature pairs. If the PCC value between two features was >0.99, one of them was removed. After this process, the dimension of the feature space was reduced, and each feature was independent of each other.

To determine the best number of features to be retained in the model, three feature selectors were compared: recursive feature elimination (RFE), analysis of variance (ANOVA), and the Kruskal–Wallis test (KW). For the classifier, we compared the performance of the linear support vector machine (SVM) and logistic regression (LR). To find the best model for each subgroup, we tested different combinations of feature selectors and classifiers and selected the combination with the best cross-validation area under the receiver operating characteristic (ROC) curve (AUC) (21, 22). We used 5-fold cross-validation to determine the hyper-parameter, which was set according to the model performance with the validation dataset. Model discrimination results were obtained by bootstrap.

**Figure 1:**
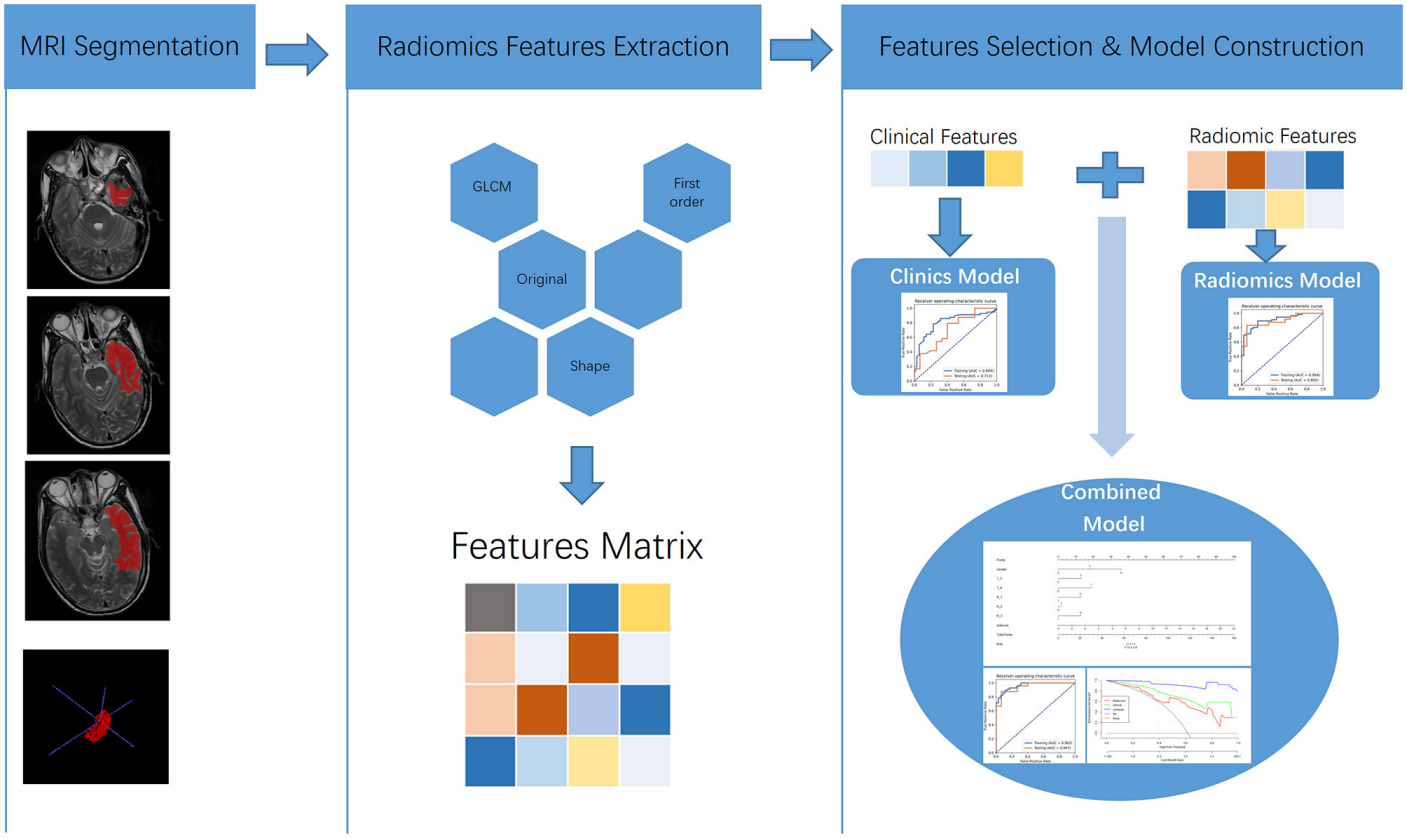
Flowchart of MRI-based predictive model construction for the occurrence of RTLI in patients with NPC.

Finally, we found that the radiomics model developed by logistic regression analysis based on six features can get the highest AUC on the validation dataset. These six features were most significantly related to RTLI. Incorporating the above-selected radiomics signatures and three independent clinical factors (gender, N stage, and T stage) by logistic regression analysis, a radiomics–clinics combined model was built using the logistic regression method (21). The combined model was visualized as a radiomics nomogram. All procedures, illustrated in Figure 1, were performed using FAE and R software.

### Statistical analysis

The statistical analyses were conducted with R software (version 4.1.0; https://www.r-project.org) and SPSS (version 20.0; IBM Corporation, Armonk, NY, USA). Differences in continuous and categorial clinical variables between the RTLI-positive and RTLI-negative groups were examined using the independent *t*-test and chi-square test, respectively. Univariate logistic regression analysis was performed to identify independent predictors among all the clinical variables. Intra-class correlation coefficients with 95% confidence intervals (CIs) were calculated to assess measure reproducibility.

The predictive ability of the models was assessed using AUCs, accuracy, specificity, and sensitivity values, and positive predictive value (PPV) and negative predictive value (NPV) from the ROC curve analysis. We also estimated 95% of CIs *via* bootstrapping with 1,000 samples. Differences among models were assessed using the DeLong test. Decision curve analysis was performed to evaluate the clinical significance of the individual predictive models.

## Results

### Patient characteristics

The sample comprised 130 patients: 80 patients with RTLI (26 bilateral and 54 unilateral; 64 men and 16 women, mean age 50.30 ± 8.86 years) and 50 patients without RTLI (31 men and 19 women, mean age 41.82 ± 9.42 years). The patient's gender and T, N, and clinical stages differed significantly between groups (*P* < 0.05; Table 1). The training and testing cohorts comprised 91 cases (56/35 = positive/negative) and 39 cases (24/15 = positive/negative), respectively. There were no significant differences in age, gender, T or N stage, pathological tumor type, radiation dose, and chemotherapy receipt between the training and testing cohorts. Univariate logistic regression analysis showed that the gender, N stage, and T stage were identified as independent clinical factors for RTLI (*P* = 0.026, 0.009, and 0.039, respectively).

**Table 1:** Sample characteristics.

**Variable**	**RTLI-positive group (*n =* 80)**	**RTLI-negative group (*n =* 50)**	***P***
Age (mean ± sd), years	50.30 ± 8.86	41.82 ± 9.42	0.798
**Gender**			0.040
Male	64	31	
Female	16	19	
**WHO pathological type**			0.089
I	0	0	
IIa	10	1	
IIb	67	48	
III	3	1	
**T stage**			0.009
T1	1	1	
T2	5	4	
T3	26	30	
T4	48	15	
**N stage**			< 0.001
N0	8	2	
N1	15	15	
N2	50	13	
N3	7	20	
**Clinical stage**			0.014
II	1	2	
III	29	20	
IVA	50	23	
IVB	0	5	
Radiotherapy dose	7,103.96 ± 136.53	7,093.24 ± 147.66	0.653

Differences between RTLI-positive and RTLI-negative groups were determined using the t-test (age) or chi-square test (classification variables).

* P < 0.05.

### Predictive performance of models

**Figure 2:**
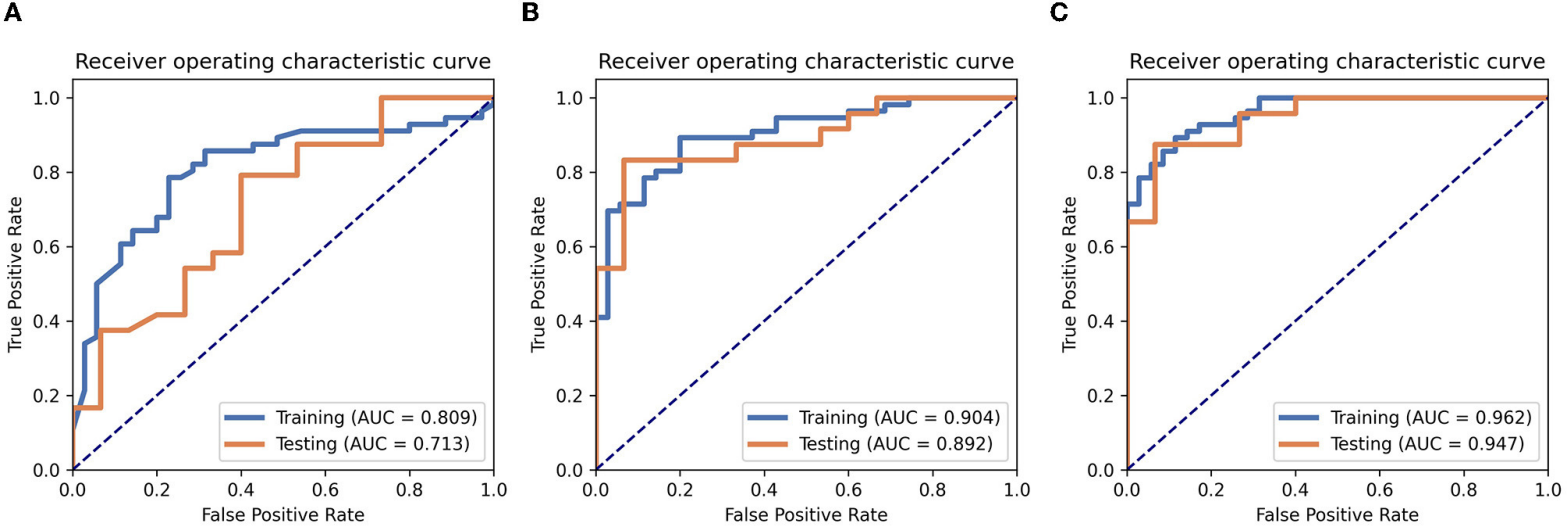
Performance analysis. The ROC analyses of the clinics **(A)**, radiomics **(B)**, and radiomics–clinics combined **(C)** models in training cohort and testing cohort.

Six texture features and three independent clinical factors (gender, N stage, and T stage) most significantly associated with early RTLI were used for building a combined model (Table 2). We demonstrated the power and potential advantages of the three models to predict the occurrence of RTLI and verified its reliability and stability. The details of the performance analysis of the three models are shown in Table 3, and the ROC curve is shown in Figure 2.

**Table 2:** The coefficients of selected features in the radiomics model.

**Features**	**Coef in model**
T1WI_original_firstorder_TotalEnergy	4.901
T1WI-CE_original_firstorder_TotalEnergy	5.081
T2WI_original_firstorder_TotalEnergy	13.506
T2WI_original_shape_Maximum2DDiameterRow	3.937
T2WI_original_shape_Sphericity	−5.918
T2WI_original_shape_SurfaceArea	2.728

**Table 3:** Model performance for the prediction of RTLI occurrence in patients with NPC.

**Model**	**AUC**	**Accuracy**	**Sen**	**Spe**	**PPV**	**NPV**
**Clinics**						
Train	0.809 (95%CI: 0.7164–0.9015)	0.780	0.785	0.771	0.846	0.692
Test	0.713 (95%CI: 0.5419–0.8831)	0.717	0.791	0.0.60	0.760	0.642
**Radiomics**						
Train	0.904 (95%CI: 0.8431–0.9651)	0.857	0.892	0.800	0.877	0.823
Test	0.891 (95%CI: 0.7903–0.9930)	0.871	0.833	0.933	0.952	0.777
**Combined**						
Train	0.962 (95%CI: 0.9306–0.9939)	0.890	0.892	0.885	0.925	0.837
Test	0.947 (95%CI: 0.8841–1.0000)	0.897	0.875	0.933	0.954	0.823

Among the three models, the combined model showed the best predictive performance with the training and testing cohorts. AUCs for the combined and radiomics models were 0.962 (95% CI, 0.9306–0.9939) and 0.904 (95% CI, 0.8431–0.9651), respectively, for the training cohort and 0.947 (95% CI, 0.8841–1.0000) and 0.891 (95% CI, 0.7903–0.9930), respectively, for the testing cohort. These values were higher than those for the clinics model (AUC = 0.809 and 0.713 for the training and testing cohorts, respectively). The combined model detected RTLI-positive in the testing cohort with greater accuracy than the clinical model did (0.897 vs. 0.717). It also performed better than the radiomics model (0.897 vs. 0.871).

**Figure 3:**
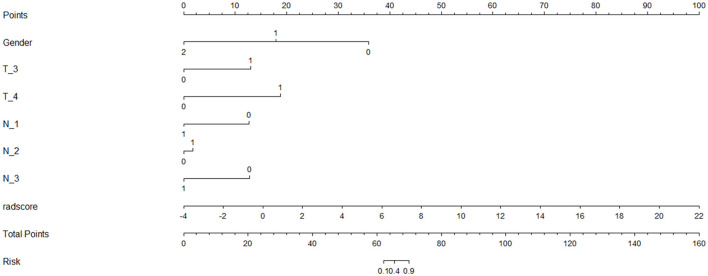
Nomogram model for the prediction of RTLI in patients with NPC.

**Figure 4:**
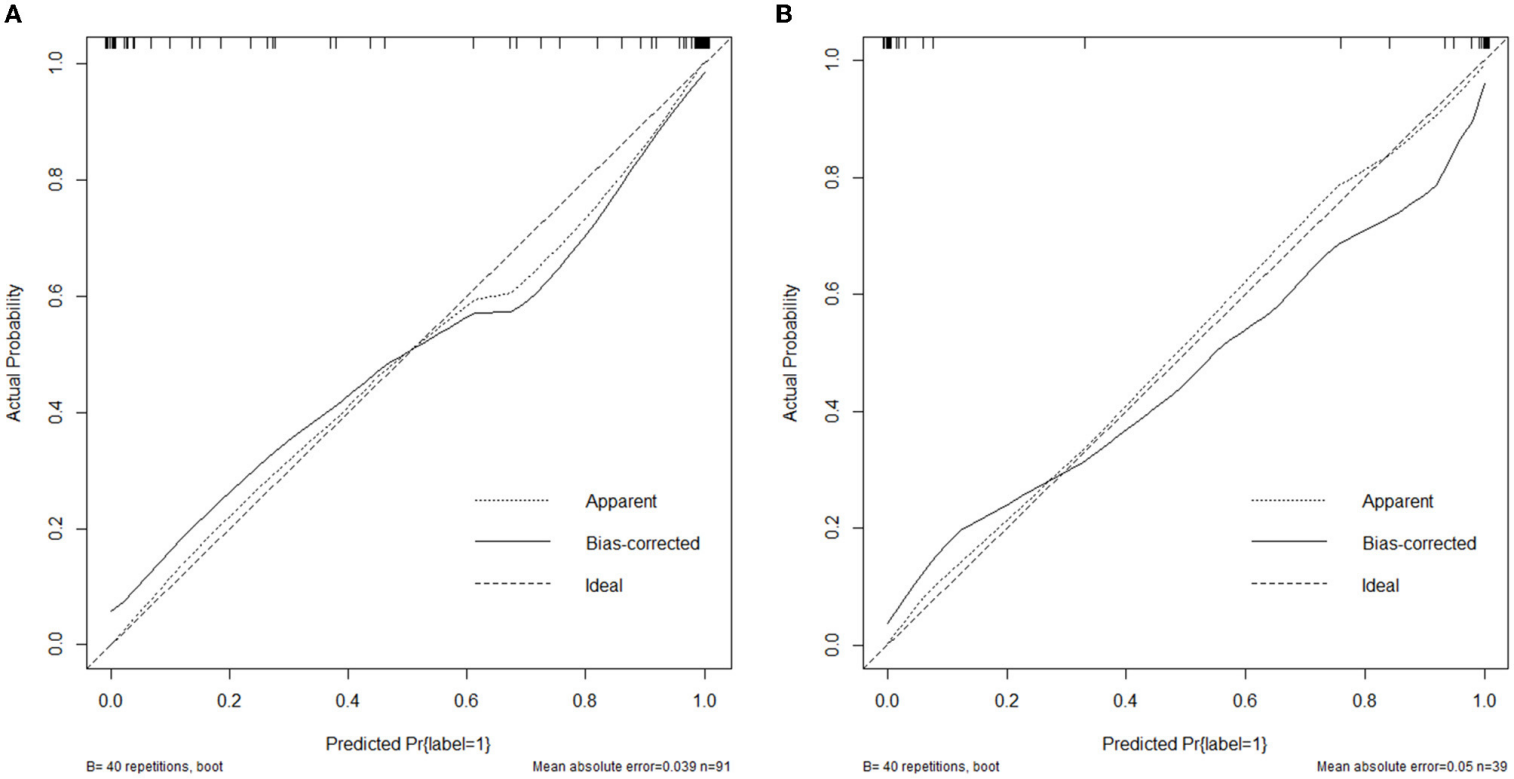
Calibration curves of the combined model developed in the training **(A)** and validation **(B)** cohorts.

**Figure 5:**
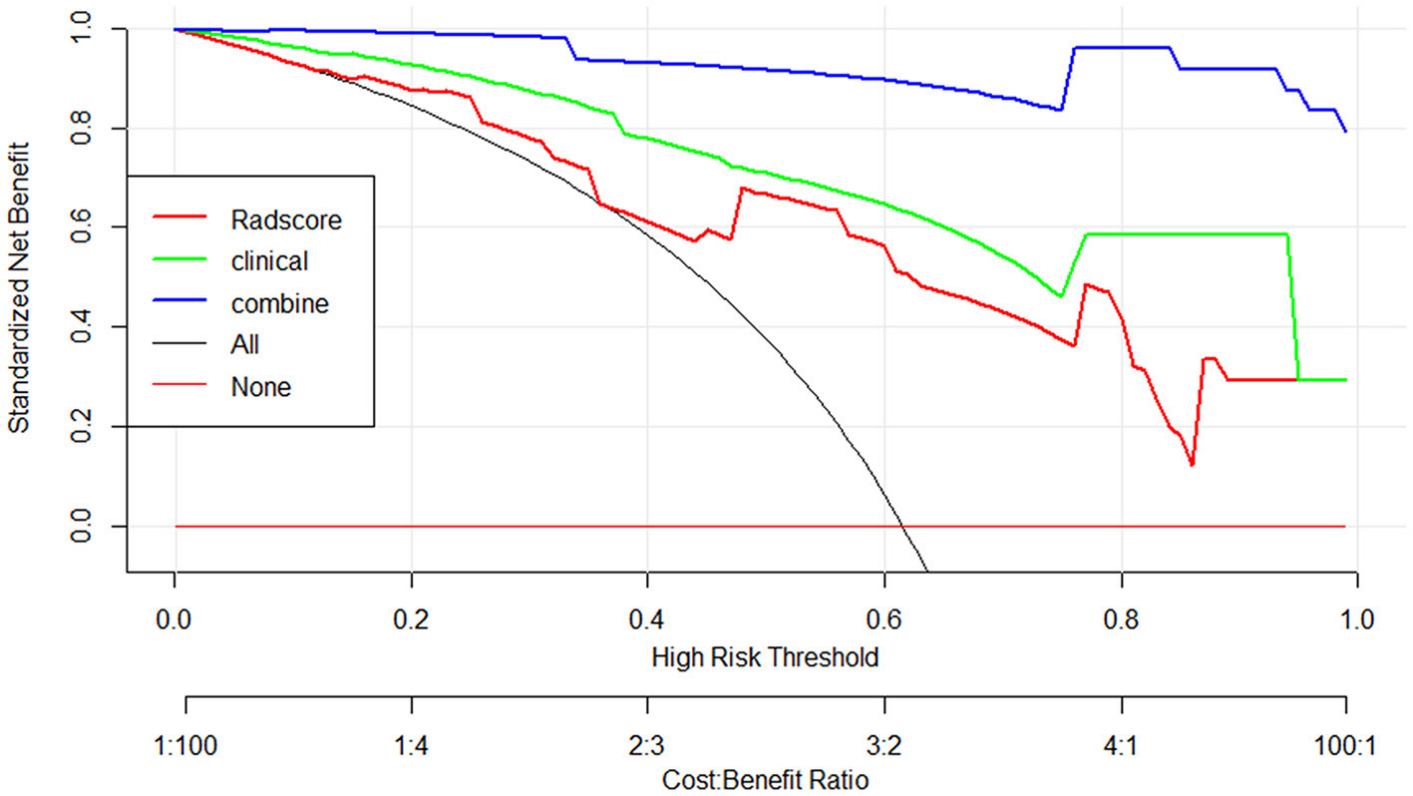
Decision curve analysis for the clinics, radiomics, and radiomics–clinics combined models for the prediction of RTLI in patients with NPC.

The nomogram that was constructed by the previously mentioned independent predictors and radiomics features is presented in Figure 3. The combined model calibration curve showed good calibration to the training and testing cohorts (Figure 4), indicating that it had good predictive power for RTLI occurrence after radiotherapy. The decision curve analysis showed that the ability of the combined model and radiomics model for predicting the occurrence of RTLI was better than that of the clinic model (Figure 5).

## Discussion

In this study, we developed and validated clinics, radiomics, and combined models for the early prediction of RTLI based on MRI data obtained at the end of IMRT courses from 130 patients with NPC with or without RTLI. The combined and radiomics models showed better predictive performance than the clinics model did for the training and testing cohorts with greater accuracy.

Few studies have examined the ability of MRI-based radiomics models to predict RTLI in the early stage. Zhang et al. (16) were the first to use MRI radiomics technology to predict RTLI. They developed three radiomics models with the AUCs of 0.872 (95% CI, 0.862–0.881), 0.836 (95% CI, 0.823–0.849), and 0.780 (95% CI, 0.759–0.800), respectively. Their findings suggest that these three radiomics models can dynamically predict RTLI in advance. However, the models did not include clinical factors related closely to RTLI, especially radiation dose parameters. Unlike the previous study, we developed the combined model for the early prediction based on the six texture features most significantly correlated with RTLI and three independent clinical factors (gender, N stage, and T stage) in our study. The combined model showed great performance in predicting RTLI with the AUC of 0.962 (95% CI, 0.9306–0.9939) and 0.947 (95% CI, 0.8841–1.0000) for the training and testing cohorts, respectively. Hou et al. (18) developed a nomogram model based on clinical factors and radiomics features for RTLI prediction; this model showed better predictive performance than radiomics and clinical factor models did. It was constructed with only texture features extracted from T2WI. In a recent study, Bao et al. (23) found that a radiomics–clinics model combining clinical T staging and radiomics features extracted from T2-weighted fat-suppressed and T1WI-CE showed a better predictive capability in RTLI than T staging alone and a single radiomics model. In this study, the texture features constructed radiomics and radiomics-clinics combined models were extracted from T1WI, T2WI, and T1WI-CE sequences.

In our study, the retaining six features that most significantly correlated with early RTLI comprised one feature extracted from T1WI sequences, one extracted from T1WI-CE sequences, and four features extracted from T2WI sequences. Some of them had greater weight than others and may reflect the brain tissue heterogeneity after radiotherapy, which may be related to the occurrence of RTLI, although the exact mechanism underlying this association is not clear. The majority of the features were extracted from T2WI sequences, in line with recent studies showing that such features have better predictive performance (16). Preclinical findings suggest that WMLs are the earliest occurring radiation injury, as the white matter is more sensitive than the gray matter to radiotherapy (24). Early pathological characteristics include increased vascular permeability, inflammatory cell infiltration, brain tissue congestion and edema, glial cell demyelination, and axonal edema. WMLs were detected best with T2WI sequences; a homogeneous increase in white matter signal intensity on these sequences is believed to represent demyelination, gliosis, and edema (25). During the development of RTLI, cerebrovascular injury and remodeling inevitably occur. T1WI-CE images reflect heterogeneity and architecture related to radiation necrosis, which in turn has been reported to correspond to focal disruption of the blood–brain barrier (24). In addition, T1WI–CE better visualizes radiation-induced hyaline degeneration in blood vessel walls, intimal reactive hyperplasia, and increased vascular permeability (26). The consideration of a combination of these texture features may, thus, improve predictive performance for RTLI.

The patient's gender, T stage, and N stage were identified as independent clinical factors for RTLI in this study. As we all know, radiotherapy planning is based on primary tumors and subclinical lesions. The temporal lobe is inevitably exposed to higher doses of radiation during treatment in patients with advanced T stage and N stage. Some scholars reported the more advanced the T grade, the greater the risk of RTLI (27). In this study, the radiation dose did not differ between RTLI-positive group and RTLI-negative group. In contrast, radiation doses were significantly higher in RTLI-positive than in RTLI-negative groups in previous studies (28–30). This difference may reflect the relationship of RTLI occurrence not only to the radiation dose but also to other factors, such as the sensitivity of the brain tissue to radiation therapy. A recent genome-wide association study implicated the genetic susceptibility gene CEP128 in RTLI development (31). In the study by Bao et al. (23), they found that T classification was the only independent clinical factor for the prediction of RTLI.

Patients with RTLI may suffer long-term headaches, insanity, dizziness, memory loss, personality changes, and seizures (32). These symptoms may greatly affect the quality of life of patients with NPC. In addition, a larger volume of the temporal lobe increased the severity of cognitive function impairment. Early detection of RTLI is helpful to reduce the incidence of temporal lobe necrosis and its related complications, such as damage to memory, language, and mobility. Recent studies have indicated that RT-induced brain injury begins in the acute period (33). A study of radiation-induced structural and functional brain abnormalities showed that early increased local brain functional activity was predictive of later severe temporal lobe necrosis (11). Thus, our attention is currently focused on early-stage RTLI. In this study, we used MRI data obtained at the end of IMRT to establish a model for the accurate and early prediction of RTLI in patients with NPC. At this point, the exogenous damage that can cause RTLI has peaked and is no longer increasing, and the brain tissue damage from the previously applied radiation may be detectable (18). Thus, our early prediction models aimed at providing a strong basis for clinicians' implementation of effective measures to prevent or slow the worsening of RTLI. Bao et al. (23) also developed a radiomics model based on MR images after the completion of radiotherapy for the prediction of RTLI and showed great performance in predicting RTLI. In addition, in the other study by Bao et al. (34), they reported that the radiomics model derived from pretreatment MRI of the temporal lobe performed well in the prediction of RTLI in patients with NPC. This is also a good starting point for predicting RTLI.

This study has several limitations. First, the RTLI-negative sample was relatively small because of the strict inclusion criteria of patients without RTLI. Second, the single-center nature of the study may limit the applicability of our findings to patients at other institutions. To extend their applicability, further external validation study is needed. Third, the temporal lobe VOIs were delineated manually. With the development of artificial intelligence (AI), automatic segmentation may become a superior approach.

## Conclusion

In conclusion, the radiomics–clinics combined model developed in this study performed well in the prediction of RTLI in patients with NPC. It may be a noninvasive and effective method for the early prediction of RTLI in patients with NPC and provide a decision-making basis for the early detection and preventive treatment of RTLI.

## Data availability statement

The raw data supporting the conclusions of this article will be made available by the authors, without undue reservation.

## Ethics statement

The studies involving human participants were reviewed and approved by the Ethics Committee of the First Affiliated Hospital of Guanxi Medical University, China. Written informed consent for participation was not required for this study in accordance with the national legislation and the institutional requirements.

## Author contributions

LH and ZY have made a substantial contribution to the concept or design of the article, the acquisition, analysis, interpretation of data for the article, and drafted the article. ZZ revised article critically for important intellectual content. HR and MJ made an analysis and interpretation of data for the article. YH and YX collected the data of all patients. HZ and KM provide software support. LL have agreed to be accountable for all aspects of the work in ensuring that questions related to the accuracy or integrity of any part of the work are appropriately investigated and resolved. All authors contributed to the article and approved the submitted version.
